# Supplementary material for: Cell divisions both challenge and refine tissue boundaries in the Drosophila embryo
Source: Development. 2026 Feb 13;153(4):dev204817. doi: 10.1242/dev.204817 (PMC12951602; doi:10.1242/dev.204817)
Supplement: Supplementary information [file develop-153-204817-s1.pdf]

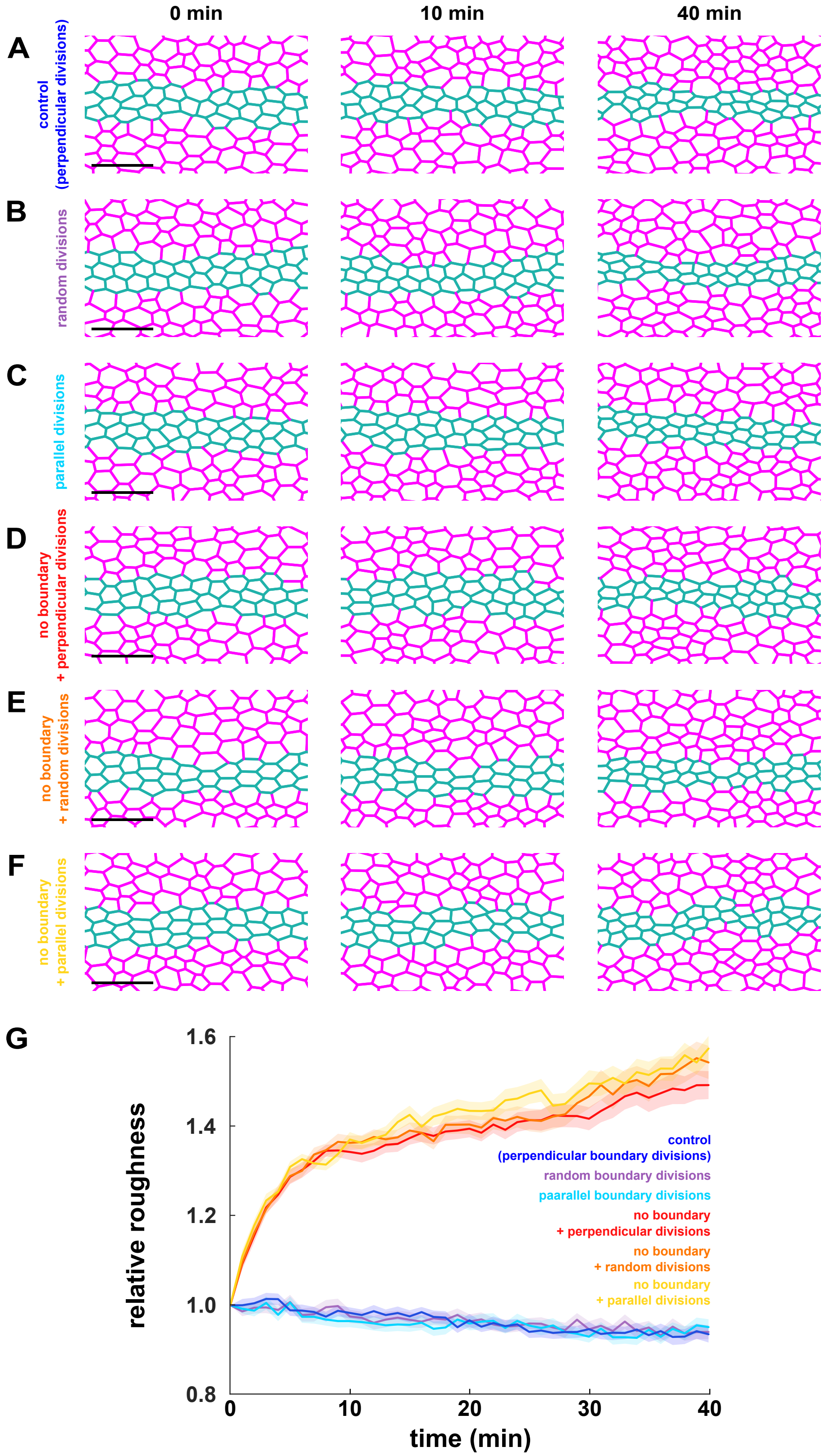
0 min
10 min
40 min
A
control
(perpendicular divisions)
B
random divisions
C
parallel divisions
D
no boundary
+ perpendicular divisions
E
no boundary
+ random divisions
F
no boundary
+ parallel divisions
G
relative roughness
time (min)
control
(perpendicular boundary divisions)
random boundary divisions
paarallel boundary divisions
no boundary
+ perpendicular divisions
no boundary
+ random divisions
no boundary
+ parallel divisions

Fig. S1. Modelling predicts that cell division orientation in ectoderm cells adjacent to the ME boundary does not challenge boundary linearity. (A-F) Simulations of mesectoderm ingression in embryos with cell divisions in ectoderm cells adjacent to the ME interface oriented perpendicular (A, D), randomly (B, E), or parallel to the interface (C, F); with (A-C) or without (D-F) tension at the boundary. Magenta, ectoderm; teal, mesectoderm. Bars, 20 µm. Anterior, left. Time zero corresponds to the time in which the mesectoderm width starts decreasing. **(G)** Relative boundary roughness in simulations with ectoderm divisions adjacent to the ME interface oriented parallel to the interface and with (blue, n = 40 simulations) or without (red, n = 40) tension at the boundary; with randomly-oriented divisions and with (purple, n = 40) and without (orange, n = 40) tension at the boundary; and with cell divisions parallel to the ME interface and with (cyan, n = 40) or without (yellow, n = 40) tension at the boundary. Error bars, s.e.m..

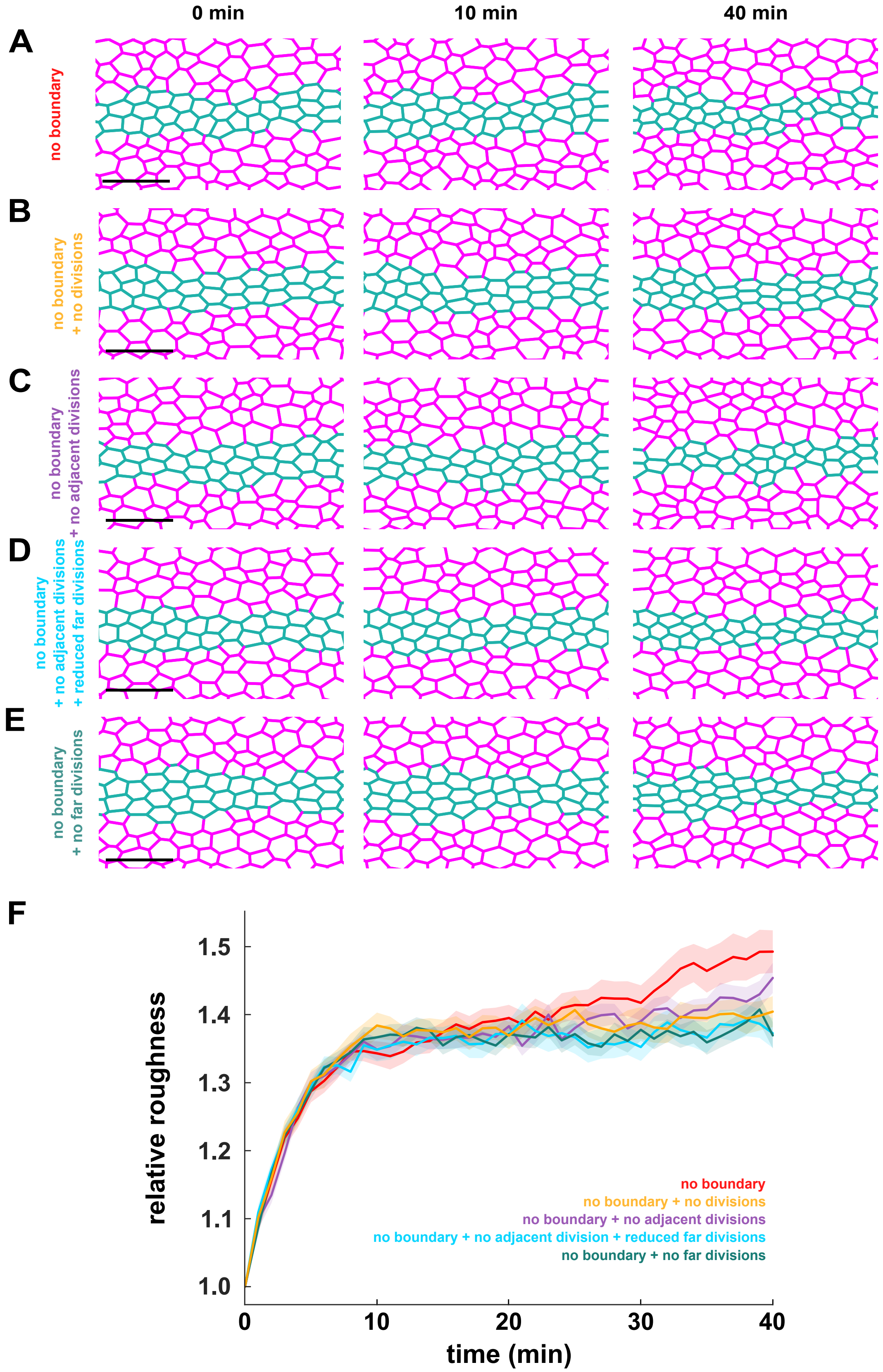
0 min
10 min
40 min
A
no boundary
B
no boundary
+ no divisions
C
no boundary
+ no adjacent divisions
D
no boundary
+ no adjacent divisions
+ reduced far divisions
E
no boundary
+ no far divisions
F
relative roughness
time (min)
no boundary
no boundary + no divisions
no boundary + no adjacent divisions
no boundary + no adjacent division + reduced far divisions
no boundary + no far divisions

Fig. S2. Cell division frequency challenges the ME boundary *in silico*. (A-E) Simulations of mesectoderm ingression in embryos with loss of tension at the ME interface (A-E) and with ectoderm divisions adjacent to and far from the ME interface (A), no ectoderm divisions (B), no ectoderm divisions adjacent to the ME interface (C), no ectoderm divisions adjacent to the ME interface and reduced divisions far from the interface (D), and no ectoderm divisions far from the ME interface (E). Magenta, ectoderm; teal, mesectoderm. Bars, 20 µm. Anterior, left. Time zero corresponds to the time in which the mesectoderm width starts decreasing. **(F)** Relative boundary roughness in simulations with loss of tension at the ME interface and: cell division adjacent to and far from the interface (red, n = 40 simulations), no ectoderm divisions (orange, n = 40), no ectoderm divisions adjacent to the interface (purple, n = 40), no ectoderm divisions adjacent to the ME interface and reduced divisions far from the interface (cyan, n = 40), and no ectoderm divisions far from the ME interface (teal, n = 40). (F) Error bars, s.e.m..

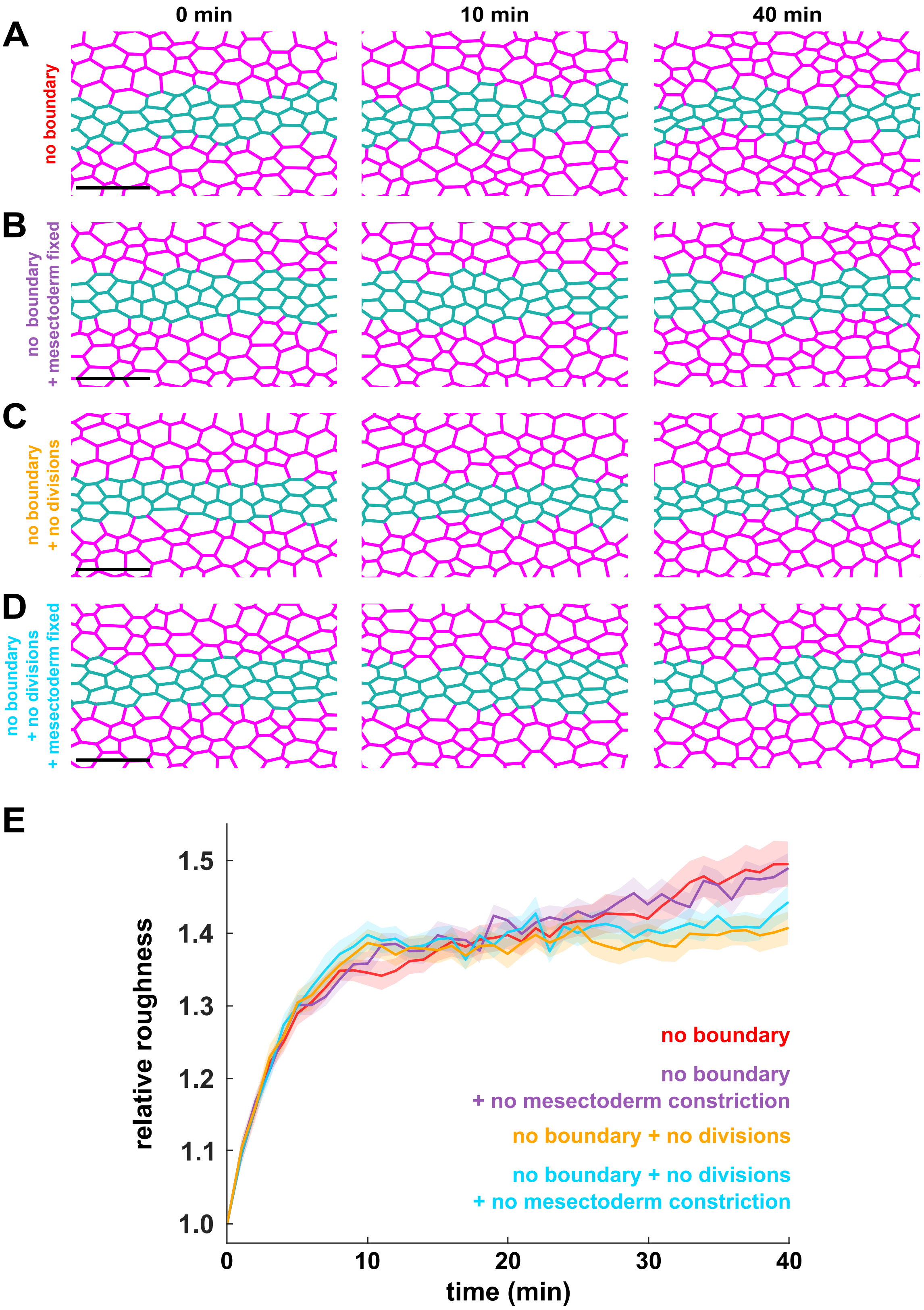

s

Development • Supplementary information

Fig. S3. Apical constriction in the mesectoderm does not challenge the ME boundary *in silico*. (A-D) Simulations of mesectoderm ingression in embryos with loss of tension at the ME interface (A-D) and with apical constriction of mesectoderm cells (A, C), no apical constriction of mesectoderm cells (B, D), cell divisions in the ectoderm (A-B) or no cell divisions in the ectoderm (C-D). Magenta, ectoderm; teal, mesectoderm. Bars, 20 µm. Anterior, left. Time zero corresponds to the time in which the mesectoderm width starts decreasing. **(E)** Relative boundary roughness in embryos with no tension at the ME interface (red, n = 40 simulations), no tension and no apical constriction of mesectoderm cells (purple, n = 40), no tension and no ectoderm divisions (orange, n = 40), or no tension, no ectoderm divisions, and no apical constriction of mesectoderm cells (cyan, n = 40). (E) Error bars, s.e.m..

Development • Supplementary information

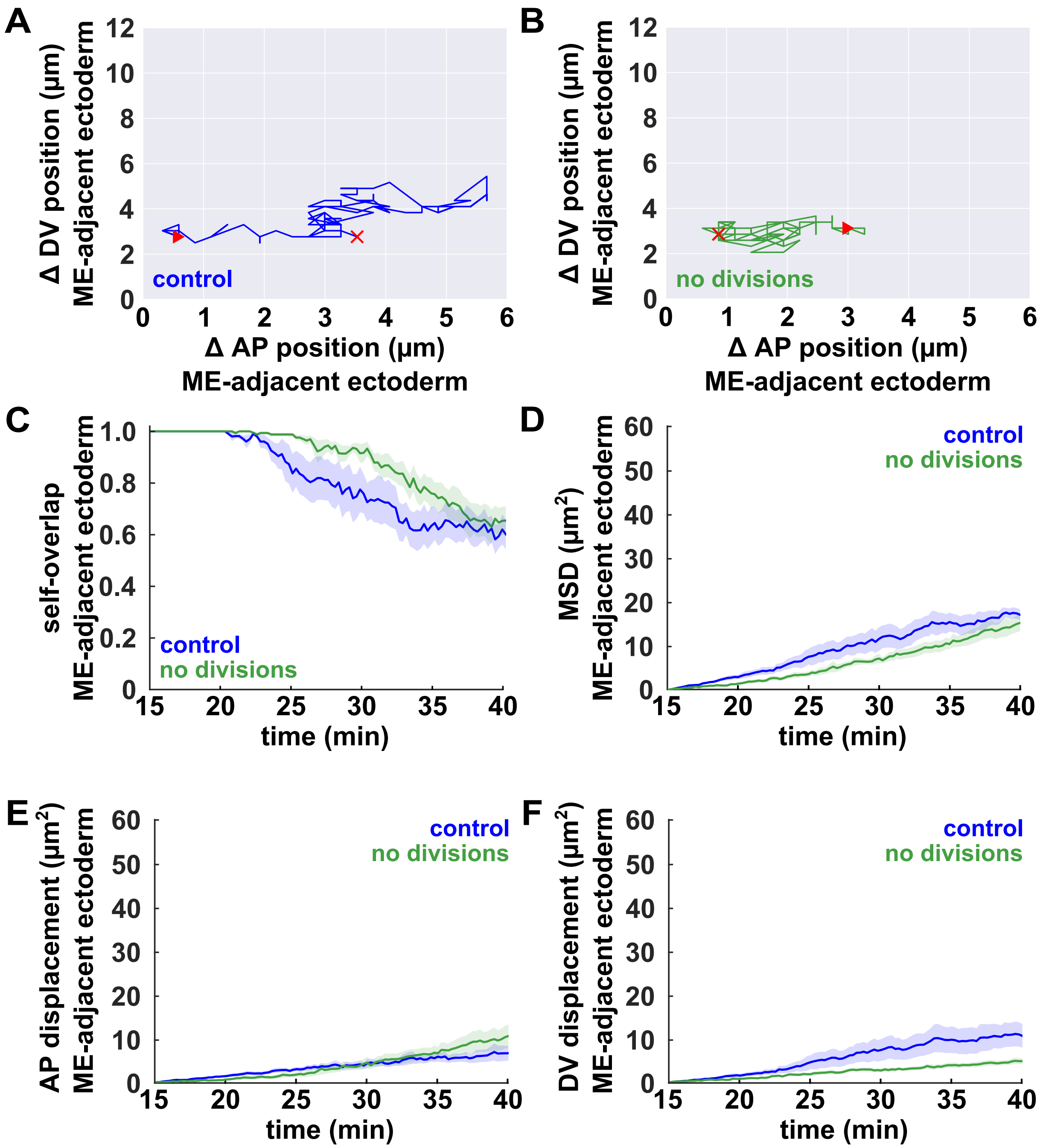

Fig. S4. Ectoderm cell divisions do not increase the mobility of ectoderm cells adjacent to the ME boundary. (A-B) Sample trajectories for ectoderm cells adjacent to the ME boundary *in vivo* for controls (blue) or when cell divisions were inhibited (green). Red triangles and crosses indicate the starting and final points of the trajectories, respectively. **(C-F)** Self-overlap function (C), MSD (D), mean squared anterior-posterior (AP) displacement (E) and mean squared dorsal-ventral (DV) displacement (F) for ectoderm cells adjacent to the ME boundary *in vivo* in DMSO-treated controls (blue, n = 5 embryos, 21-30 cells per embryo) and in dinaciclib-treated embryos (green, n = 6 embryos, 24-32 cells per embryo). Error bars, s.e.m..

Development • Supplementary information

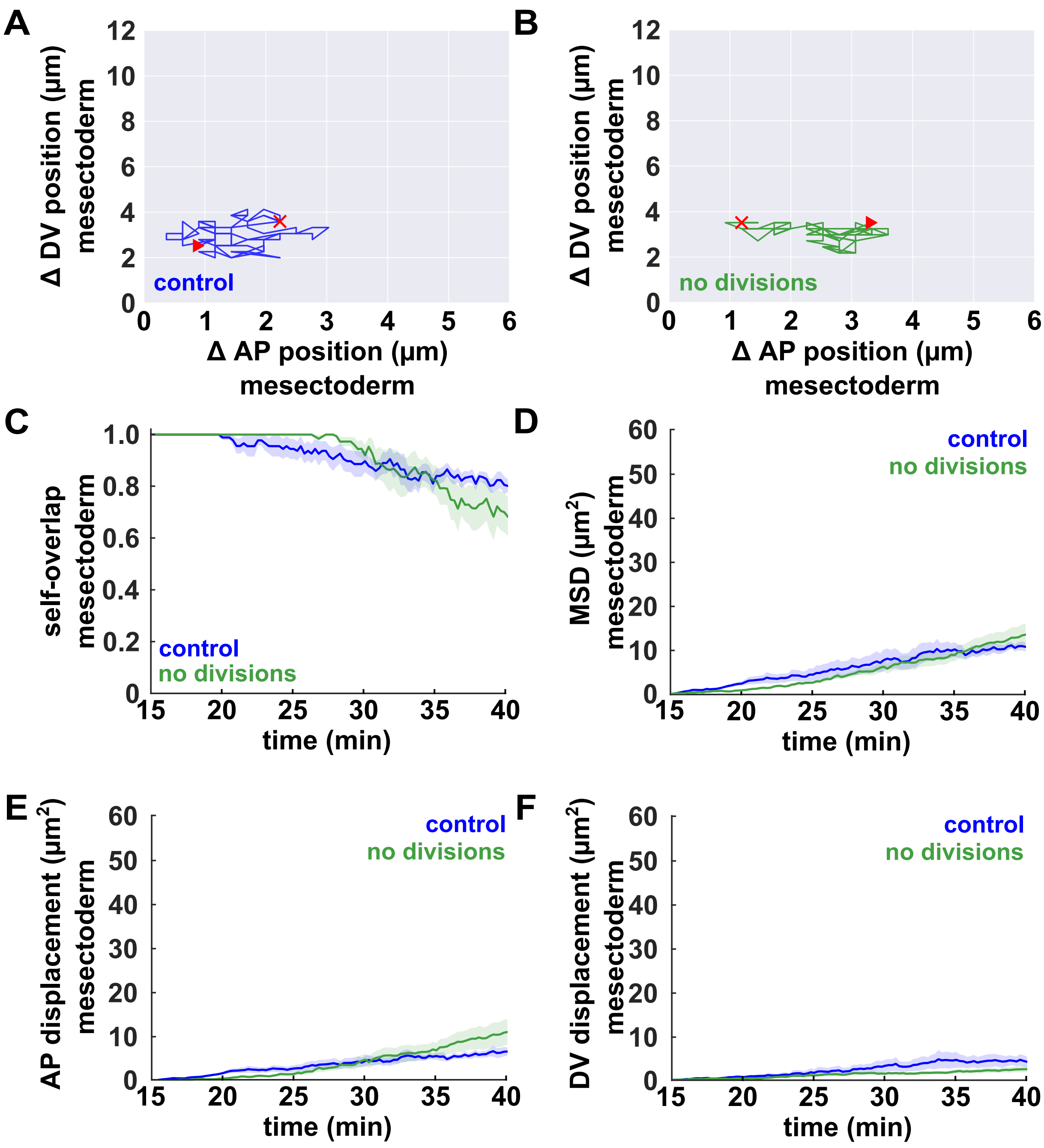

Fig. S5. Ectoderm cell divisions do not fluidize the mesectoderm. (A-B) Sample mesectoderm cell centroid trajectories *in vivo* for controls (blue) or when cell divisions were inhibited (green). Red triangles and crosses indicate the starting and final points of the trajectories, respectively. **(C-F)** Self-overlap function (C), MSD (D), mean squared anterior-posterior (AP) displacement (E) and mean squared dorsal-ventral (DV) displacement (F) for mesectoderm cells *in vivo* in DMSO-treated controls (blue, n = 5 embryos, 18-27 cells per embryo) and in dinaciclib-treated embryos (green, n = 6 embryos, 19-26 cells per embryo). Error bars, s.e.m..

Development • Supplementary information

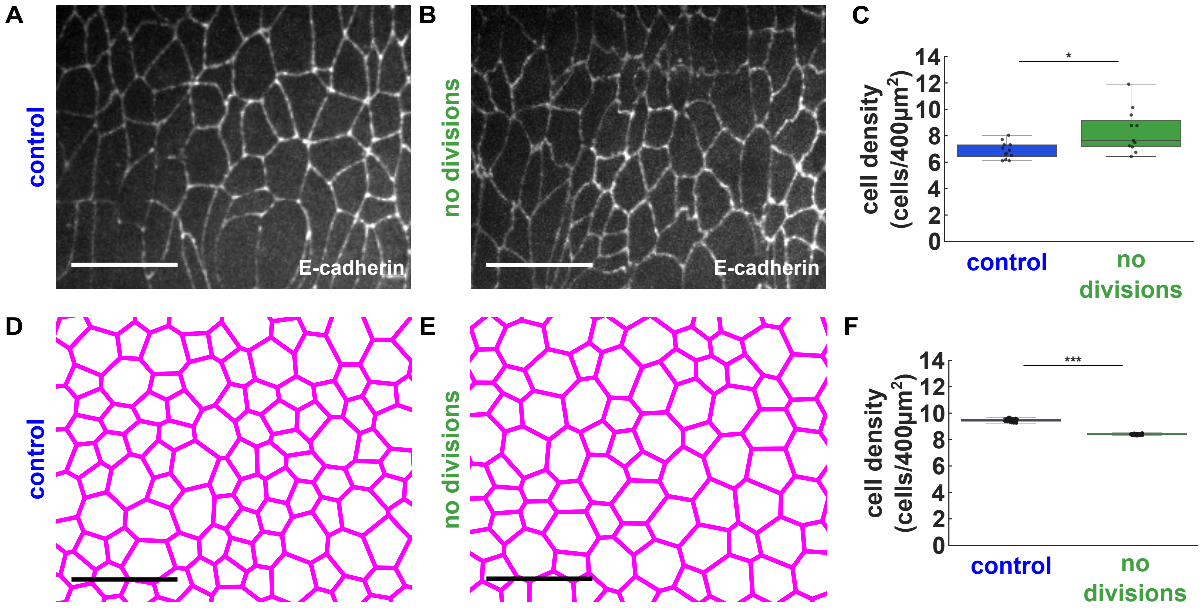

Fig. S6. Inhibiting cell division has different effects on ectoderm cell density *in vivo* and *in silico*. (A-B) Ectoderm cells expressing E-cadherin:GFP, in embryos treated with 50% DMSO (A) or 500 µM dinaciclib (B) 30 min after treatment. Bars, 20 µm. Anterior, left; ventral, down. **(C)** Ectoderm cell density 30 min after treatment for embryos injected with 50% DMSO (blue, n = 12 embryos) or with 500 µM dinaciclib (green, n = 11). **(D-E)** Ectoderm cells in simulations of mesectoderm ingression in controls (D) or when ectoderm cell divisions were inhibited (E). **(F)** Ectoderm cell density in control simulations (blue, n = 80 simulations), or in simulations with no cell divisions (green, n = 80) 30 min after inhibiting cell division. Error bars, s.e.m.. Error bars, range; box, quartiles; grey lines, median. * $P < 0.05$, *** $P < 0.001$.

Development • Supplementary information

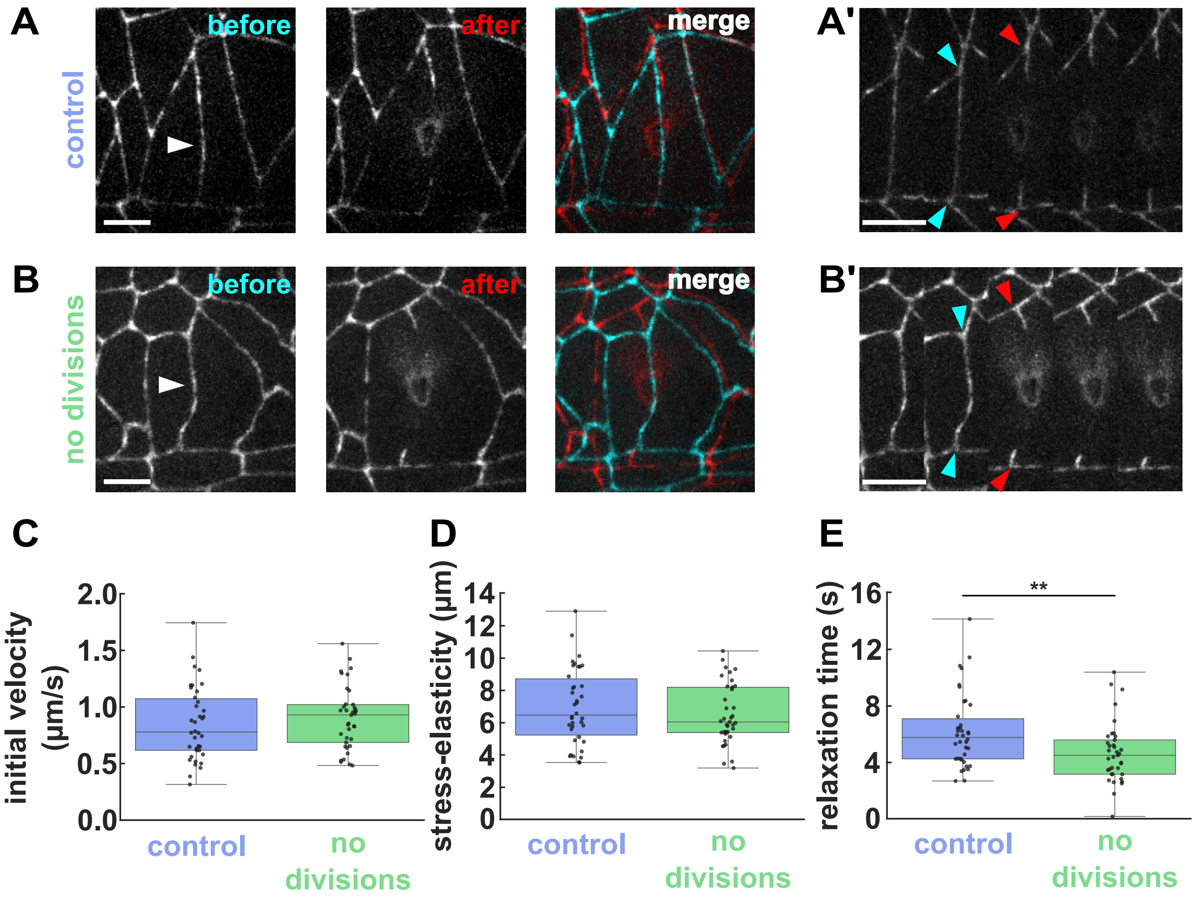

Fig. S7. Inhibiting cell division does not affect ectoderm tension adjacent to the mesectoderm. (A-B) Ectoderm cells adjacent to the ME boundary and expressing E-cadherin:GFP, immediately before (left, cyan in merge) and after (right, red in merge) ablation of a cell-cell junction parallel to the dorsal-ventral axis, in embryos treated with 50% DMSO (A) or 500 µM dinaciclib (B). Corresponding kymographs are shown (A'-B'). Arrowheads indicate the severed interface (white, A-B), or its ends prior to ablation (cyan, A'-B') or immediately after (red, A'-B'). Bars, 5 µm (A-B) and 4 s (A'-E'). Anterior, left. **(C-E)** Initial recoil velocity after ablation (C), stress-elasticity ratio (D), and relaxation time (E) for cuts in embryos treated with 50% DMSO (blue, n = 38 cuts) or 500 µM dinaciclib (green, n = 37). (C) Error bars, s.e.m.. Error bars, range; box, quartiles; grey lines, median. ** $P < 0.01$.

Development • Supplementary information

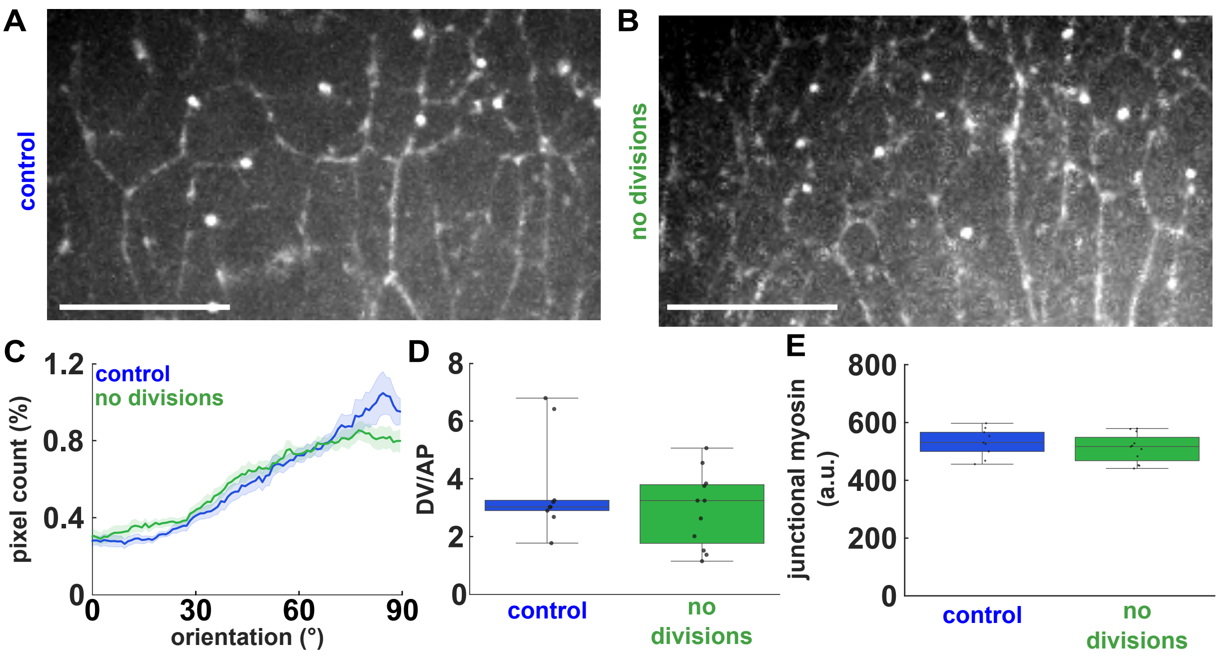

Fig. S8. Inhibiting cell division does not affect myosin distribution or levels in the ectoderm. (A-B) Ectoderm cells expressing myosin:GFP in embryos treated with 50% DMSO (A) or 500 µM dinaciclib (B). Bars, 20 µm. Anterior, left; ventral, down. **(C-E)** Histogram of gradient orientations (C), ratio of dorsal-ventral (DV) *vs.* anterior-posterior (AP)-oriented pixels (D), and junctional myosin fluorescence (E) for embryos treated with 50% DMSO (blue, n = 9 embryos) or with 500 µM dinaciclib (green, n = 11). (C). Error bars, s.e.m.. (D-E) Error bars, range; box, quartiles; grey lines, median.

Development • Supplementary information

Table S1. Simulation parameters in natural units

area spring constant, K_A	1
perimeter spring constant, K_P	1
inverse friction coefficient, μ	1
temperature, T	0.01
initial preferred area of mesectoderm cells, $A_{0mesec}^{initial}$	0.79
preferred area of ectoderm cells before division, $A_{0ecto}^{pre-division}$	1.32
preferred area of ectoderm cells after division, $A_{0ecto}^{post-division}$	0.66
target shape index of mesectoderm cells, q_{mesec}	3.4
target shape index of ectoderm cells, q_{ecto}	3.4
cell division time, t_{cd}	2
tension constant, γ_0	0.4
rate of acute tension reduction, $k_\gamma^{no\ boundary}$	0.36
rate of tension reduction representing control embryos, $k_\gamma^{control}$	0.01

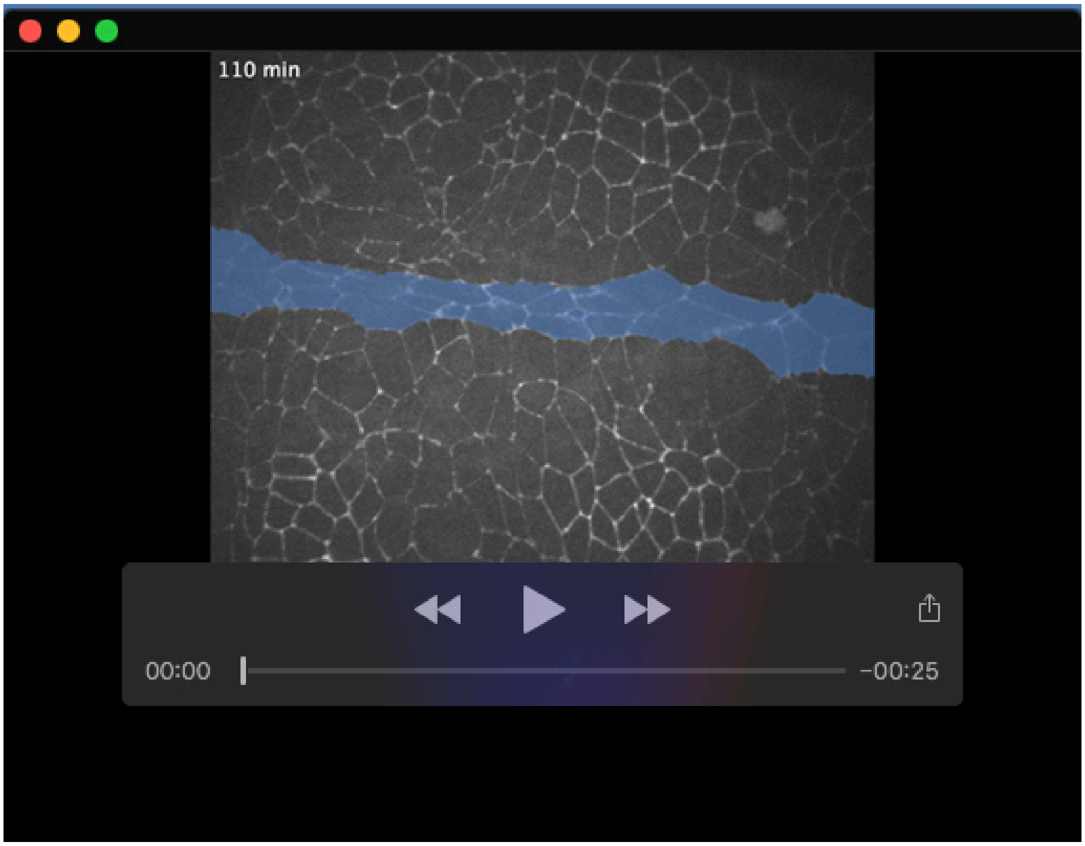

Movie 1. The mesectoderm separates the mesoderm (ventral) from the ectoderm (lateral) on both sides of the ventral midline. Mesectoderm and ectoderm cells in a *Drosophila* embryo expressing E-cadherin:GFP. Mesectoderm cells are highlighted in blue. Time is with respect to the completion of mesectoderm divisions. Anterior, left. Images were acquired every 5 minutes.

Development • Supplementary information

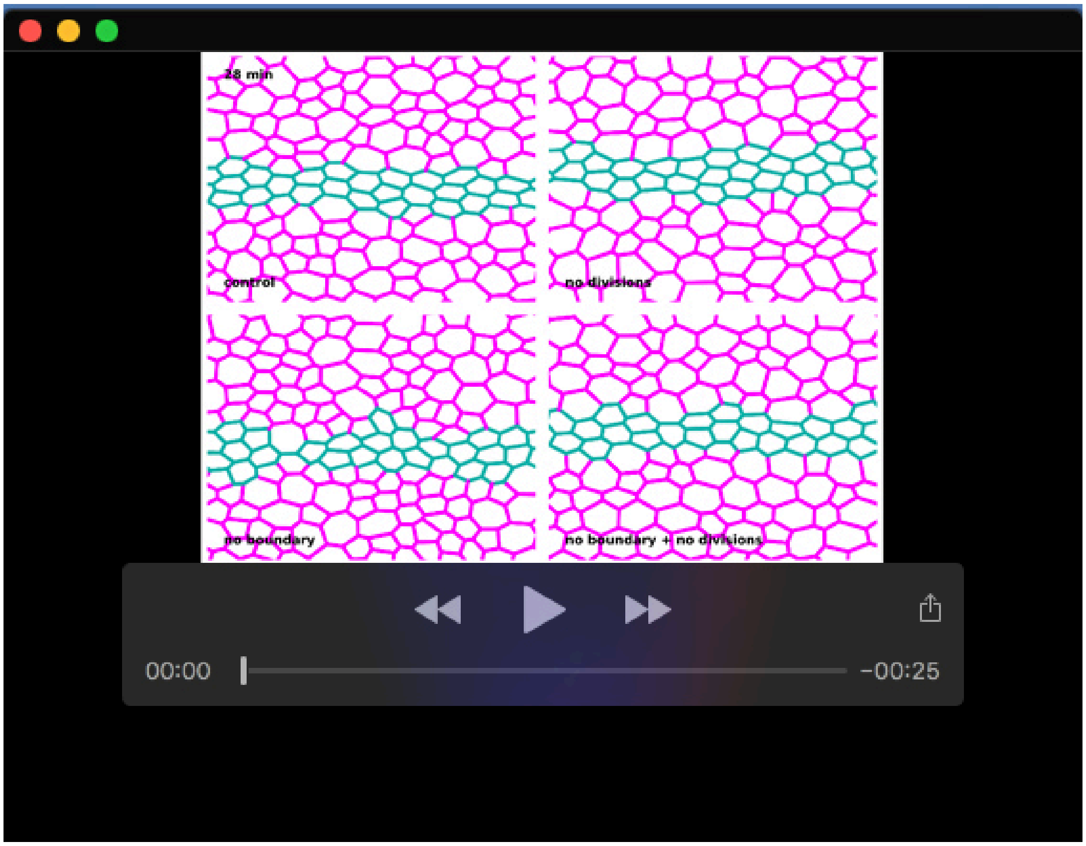

Movie 2. Mathematical modelling predicts that ectoderm divisions both challenge and refine the ME boundary. Simulations of mesectoderm ingression in control embryos (top left), with an acute loss of tension at the ME interface (bottom left), without ectoderm cell divisions (top right), or with simultaneous acute loss of tension at the ME interface and inhibition of ectoderm cell divisions (bottom right). Magenta, ectoderm; teal, mesectoderm. Anterior, left. Time zero corresponds to the time in which the mesectoderm width starts decreasing. Images were generated every 10 minutes.

Development • Supplementary information

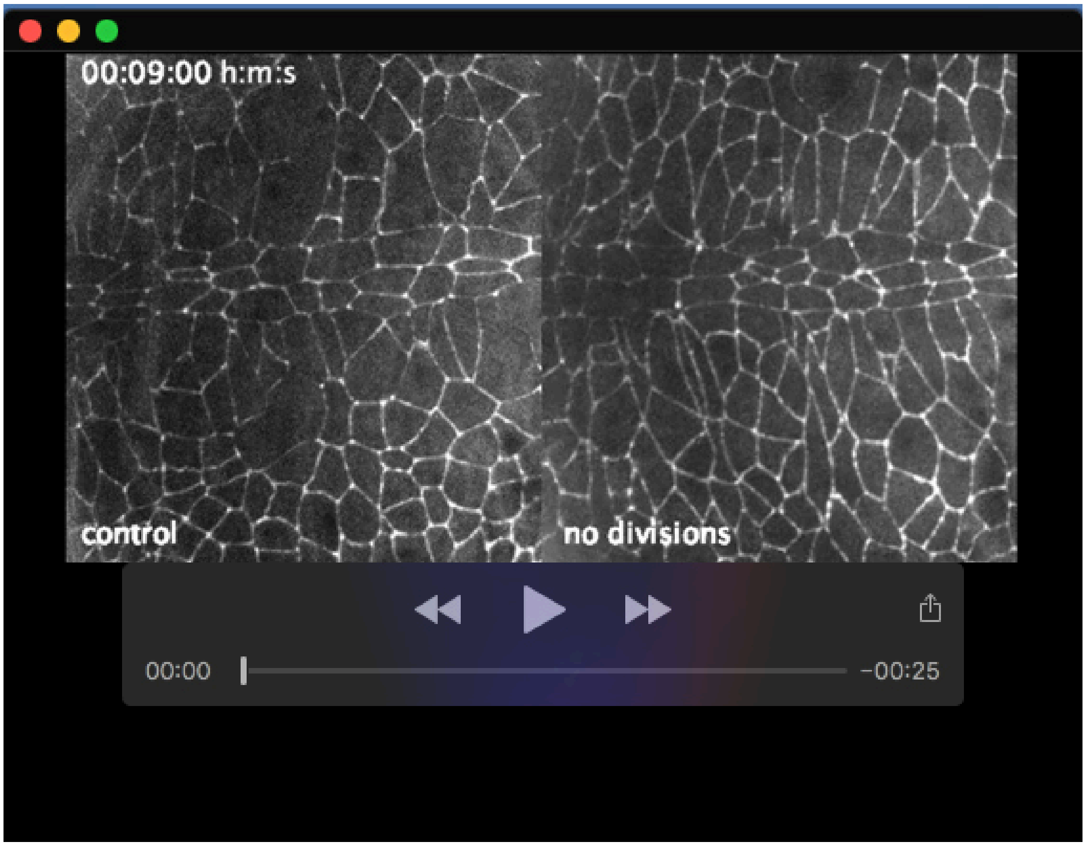

Movie 3. Dinaciclib treatment inhibits ectoderm divisions and prevents ME boundary refinement. Mesectoderm (centre) and ectoderm (top and bottom) cells in embryos expressing E-cadherin:GFP, and injected, 1 hour after the onset of mesectoderm divisions, with 50% DMSO (left) or 500 µM dinaciclib (right). Anterior, left. Images were acquired every 30 seconds.

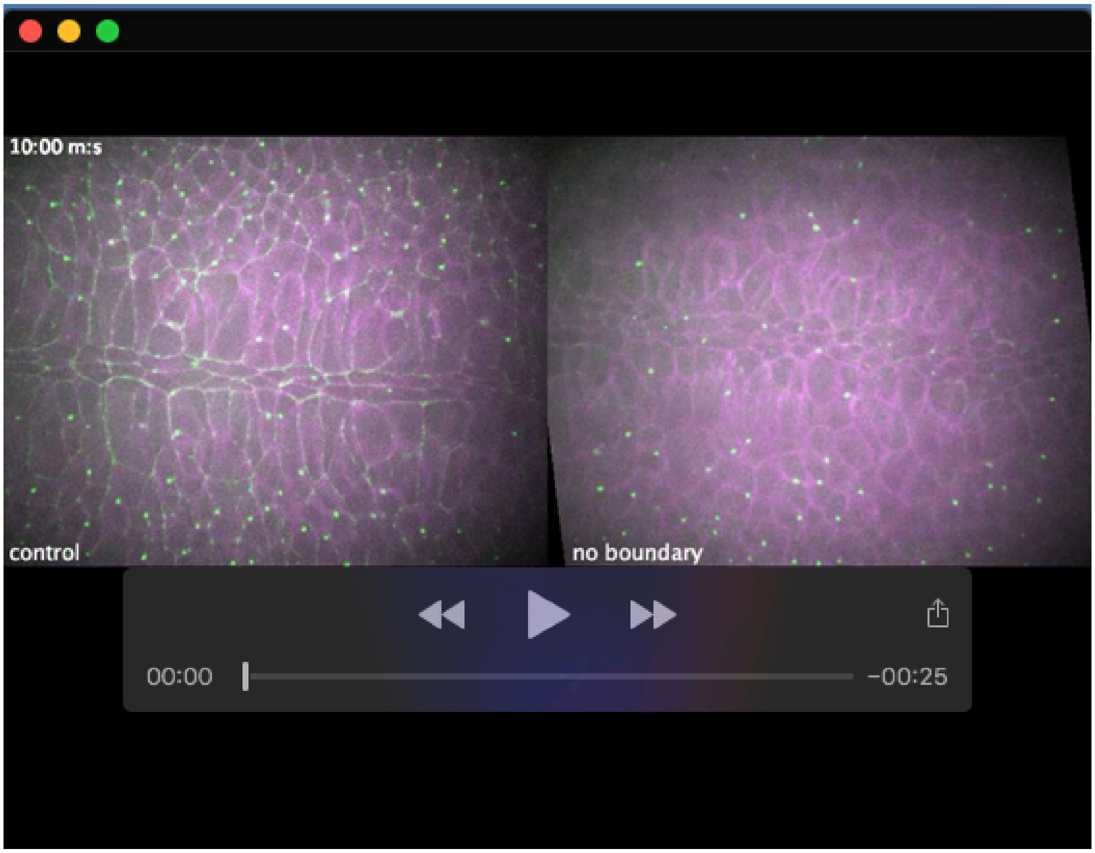

Movie 4. Y-27632 treatment does not inhibit ectoderm divisions. Mesectoderm (centre) and ectoderm (top and bottom) cells in embryos expressing myosin:GFP (green) and Gap43:mCherry (magenta), and injected, 1 hour after the onset of mesectoderm divisions, with 50% DMSO (left) or 20 mM Y-27632 (right). Anterior, left. Images were acquired every 30 seconds.

Development • Supplementary information

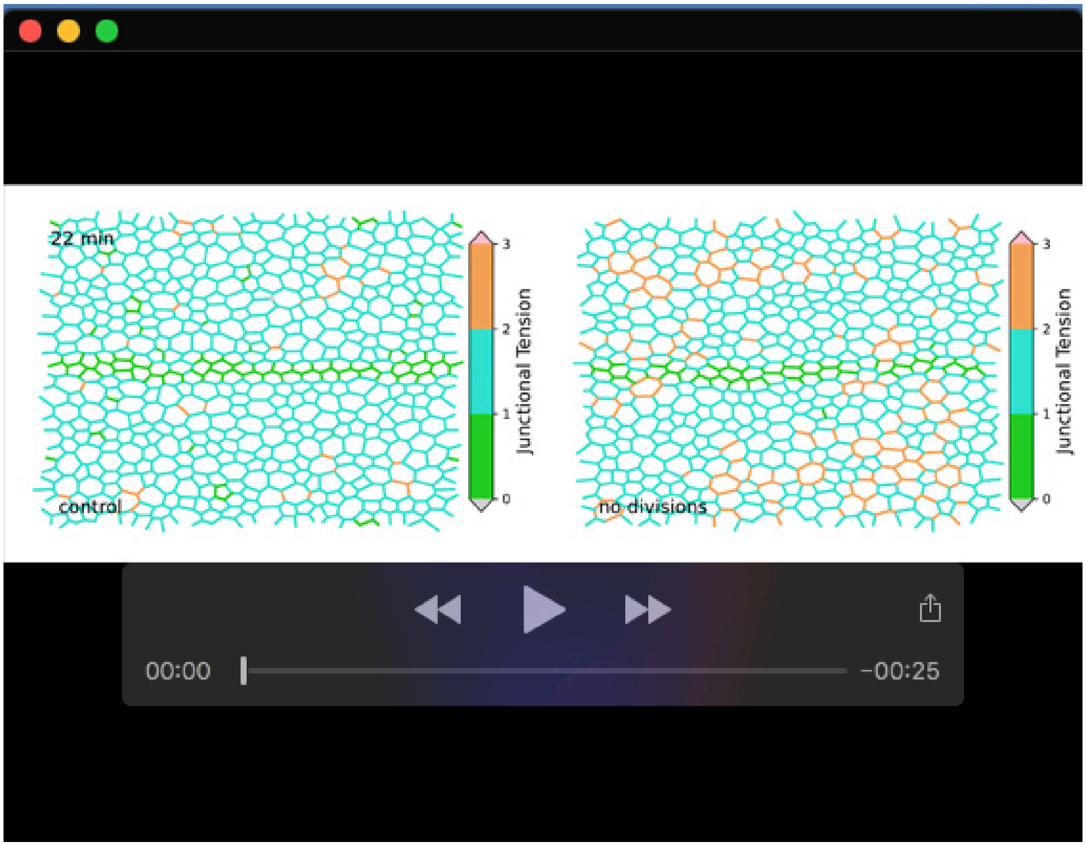

Movie 5. Mathematical modelling predicts that cell divisions reduce junctional tension in the ectoderm. Junctional tension distribution in simulations of mesectoderm ingression in controls (left) or when ectoderm cell divisions were inhibited (right). Anterior left. Time zero corresponds to the time in which the mesectoderm width starts decreasing. Images were generated every 10 minutes.

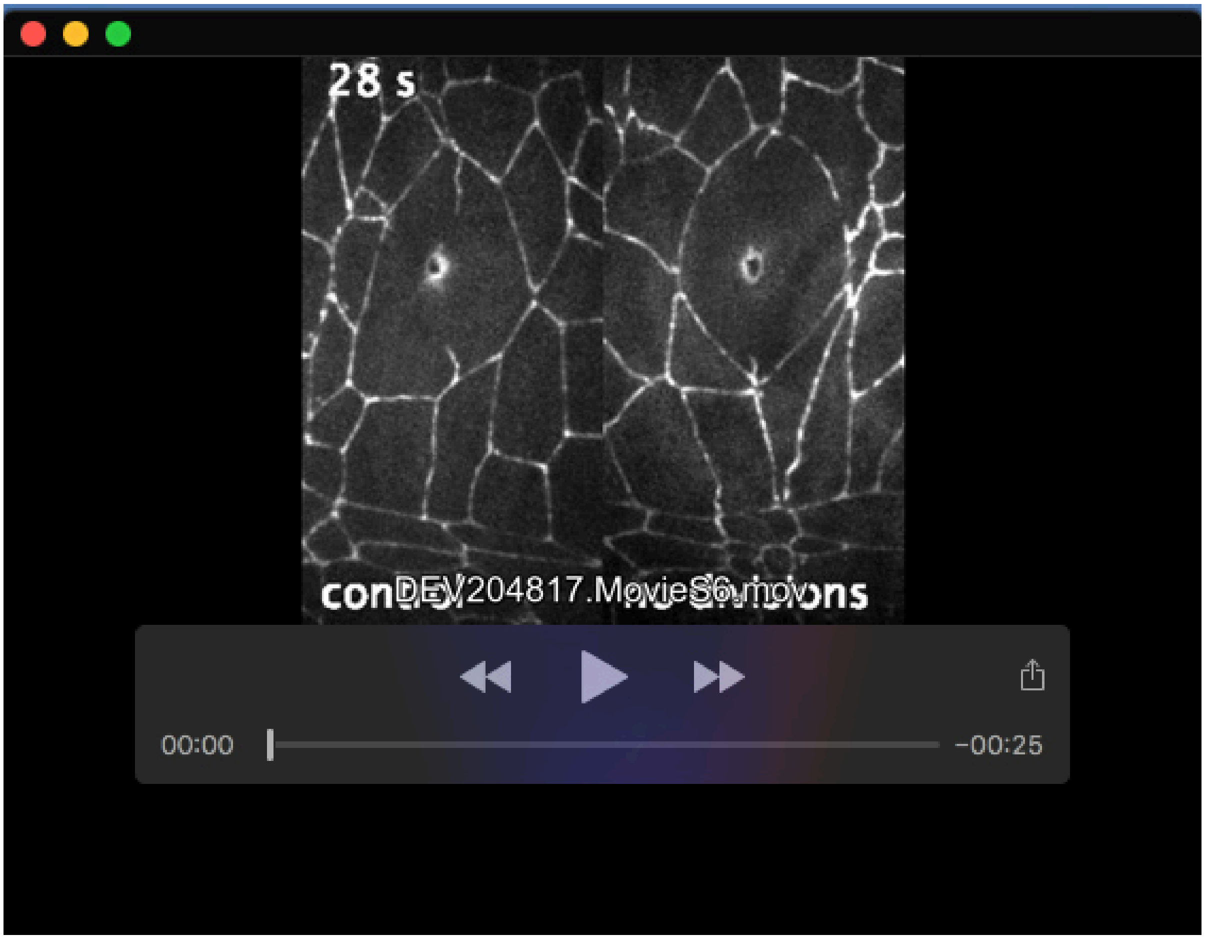

Movie 6. Cell divisions reduce junctional tension in the ectoderm. Laser ablation of a contact between ectoderm cells and parallel to the dorsal ventral axis in embryos expressing E-cadherin:GFP and treated with 50% DMSO (left) or 500 µM dinaciclib (right). Images were acquired every 4 seconds.
